# Tuning Lewis Acidity in MXene-Supported Single-Atom Catalysts

**DOI:** 10.3390/nano16130800

**Published:** 2026-06-27

**Authors:** Weiqiang Sun, Tingting Zhou, Boyu Han, Hu Xu, Junqi Wang, Bo Yu

**Affiliations:** 1 School of Nuclear Science and Technology, Xi’an Jiaotong University, Xi’an 710049, China; sunweiqiang@xjtu.edu.cn; 2 School of Human Settlements and Civil Engineering, Xi’an Jiaotong University, Xi’an 710049, China; 372498563@stu.xjtu.edu.cn (T.Z.); hanboyu0036@stu.xjtu.edu.cn (B.H.); 3 School of Physics, Xi’an Jiaotong University, Xi’an 710049, China; xu_hu@xjtu.edu.cn; 4 State Key Laboratory of Advanced Casting Technologies, Shenyang 110022, China

**Keywords:** MXene, single-atom catalyst, Lewis acidity, biomass conversion

## Abstract

Regulation of surface acidity is critical for steering reaction pathways in catalytic biomass conversion; however, the modulation of Lewis acidity in MXene-supported single-atom catalysts remains poorly understood. In this work, density functional theory calculations were performed to systematically investigate how surface terminations coupled with metal identity govern the Lewis acidity of single-atom sites on Ti_3_C_2_-based MXenes. Two representative terminations (-O and -OH) were considered, and various metal atoms were anchored to construct single-atom catalysts. Formation energies were evaluated to assess thermodynamic stability, while NH_3_ adsorption energies were employed as a descriptor for Lewis acidity. The results reveal a pronounced termination-dependent modulation of acidity. Specifically, -OH termination disfavors single-atom stabilization, whereas -O termination ensures strong anchoring. Electronic structure analysis indicates that enhanced acidity originates from termination-induced electronic polarization and charge redistribution. This work establishes a structure–termination–acidity relationship and provides theoretical guidance for the rational design of MXene-based catalysts with tunable acidity.

## 1. Introduction

Biomass is abundant, renewable, and possesses a near carbon-neutral potential, and is therefore widely regarded as a promising alternative to fossil resources for enabling resource recycling and sustainable chemical production. The efficient conversion of biomass into fuels and value-added chemicals can not only reduce dependence on non-renewable feedstock but also provide a viable pathway toward a sustainable chemical industry [1]. However, biomass compounds—especially typical components such as cellulose—are highly oxygenated and structurally complex, featuring diverse reaction pathways. As a result, thermochemical conversion of biomass often involves multiple competing reactions and produces complex product distributions, making it challenging to enhance target selectivity and achieve process controllability [2,3]. Therefore, developing efficient catalysts capable of steering reaction pathways in a controllable manner is crucial for improving both the conversion efficiency and product selectivity in biomass valorization.

In a wide range of biomass conversion processes, catalytic acidic sites are generally recognized as key factors governing substrate adsorption configurations, activation of critical chemical bonds, and stabilization of reaction intermediates. Acidic sites can interact with oxygen-containing functional groups in biomass-derived substrates, thereby regulating key steps such as depolymerization and dehydration and ultimately shaping product distributions [4,5]. To construct and tailor acidic sites, various catalytic systems have been developed, including liquid acids and solid acid catalysts, as well as acidity-containing materials such as metal oxides, zeolites, and carbon-based materials [6,7,8]. In general, solid acid catalysts exhibit superior recyclability, stability, and sustainability, making them more favorable for scalable and green processes. Nevertheless, the type, strength, and spatial distribution of acidic sites in solid catalysts are often constrained by the support structure and surface chemistry, highlighting the need for more refined catalyst-design strategies to achieve pathway-selective biomass conversion [9,10].

MXenes (M_n+1_X_n_T*_x_*) represent an emerging family of two-dimensional materials featuring high surface area, tunable surface terminations, and excellent interfacial designability, offering a unique platform for constructing adjustable acidic sites [11,12]. Based on our previous studies, MXenes have demonstrated considerable catalytic activity in thermocatalytic biomass conversion, suggesting their potential as novel solid-acid or interface-tailored catalytic materials. Previous studies have shown that surface terminations on MXenes are strongly influenced by the underlying metal framework, leading to effective modulation of surface acidic sites and catalytic behavior [13]. The interaction between metal centers and functional groups alters the electronic properties of surface terminations, thereby tuning acidity and lowering the energy barriers of key thermochemical steps during biomass conversion. From a catalytic standpoint, such acidity regulation is critical for controlling dehydration reactions that govern the formation of dehydrated sugar products, such as levoglucosan.

Based on this understanding, the present work systematically investigates how surface functional groups coupled with different metal atoms regulate the Lewis acidity of MXene-supported single-atom sites and thereby influence catalytic behavior. Ti-based MXene was selected as the model substrate, and two representative surface terminations (-OH and -O) were considered. Single-atom catalysts were constructed by anchoring various metal atoms (Ti, Ni, Fe, Ga, Sn, and Ru) onto these functionalized MXene surfaces. Density functional theory calculations were employed to evaluate the formation feasibility and stability of single-atom sites, while NH_3_ adsorption energy was used as a descriptor to quantify Lewis acidity. Electronic structure analyses, including density of states and charge population analysis, were further performed to elucidate the origin of termination-dependent acidity.

## 2. Method

### 2.1. Density Functional Theory Calculations

**Figure 1:**
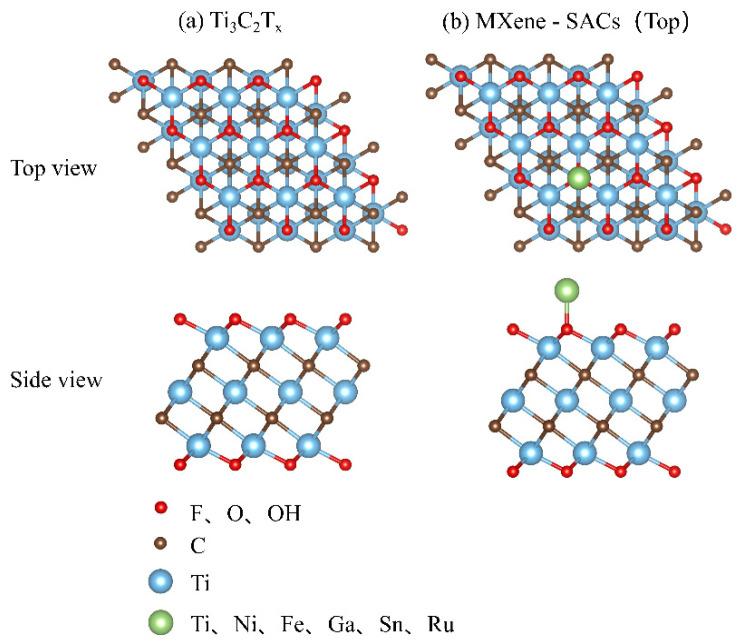
Schematic of Ti_3_C_2_ MXene pristine and doped monolayers.

Density functional theory (DFT) calculations were performed using the CASTEP module. A Ti_3_C_2_-based MXene slab model was constructed to represent the functionalized MXene surface, and single metal atoms were adsorbed on the top sites of surface functional groups to form isolated single-atom configurations (Figure 1). The metal atoms were initially placed at the termination-top sites according to commonly adopted MXene-supported SAC models [14,15], allowing a unified comparison of termination-dependent electronic effects and Lewis acidity. All pristine and metal-anchored MXene structures were fully optimized prior to property analysis.

The electronic exchange-correlation interactions were described using the generalized gradient approximation (GGA) with the Perdew–Burke–Ernzerhof (PBE) functional. Core-valence interactions were treated using ultrasoft pseudopotentials provided within the CASTEP library. A vacuum layer of at least 15 Å was introduced along the surface normal (z-direction) to eliminate spurious interactions between periodic images. The plane-wave kinetic energy cutoff was set to 489.8 eV. Brillouin zone sampling was carried out using a 3 × 3 × 1 Monkhorst–Pack k-point mesh. Geometry optimizations were performed until the total energy change per atom was less than 2.0 × 10^−5^ eV, and the maximum residual force on each atom was below 0.05 eV/Å. The self-consistent field (SCF) convergence tolerance was set to 2.0 × 10^−6^ eV per atom. For electronic structure analysis, density of states (DOS) and partial density of states (PDOS) calculations were performed based on the optimized structures. To ensure the reliability of the electronic properties, a denser 6 × 6 × 1 k-point mesh was employed for all DOS and PDOS calculations. All calculations for different metal species and surface terminations were conducted using identical computational parameters to ensure the consistency and comparability of the calculated structural and electronic properties.

Initial molecular structures were constructed using the Amorphous Cell module. The system density was set to 1.0 g/cm^3^, and multiple initial configurations were generated to sufficiently sample the configurational space. The resulting structures were first optimized using the universal force field, and representative low-energy conformations were selected based on total energy ranking for subsequent calculations.

Subsequently, density functional tight-binding calculations were performed using DFTB+ to further optimize the geometries of the reactants, intermediates, and products. The reaction pathway was constructed based on previously reported cellulose pyrolysis mechanisms. Constrained geometry optimizations were carried out along predefined reaction coordinates, in which key bond-forming and bond-breaking distances were systematically varied to obtain the relative energy profile. The tiorg Slater–Koster parameter set was employed, and the self-consistent charge tolerance was set to 1.0 × 10^−4^. After geometry optimization, single-point energy calculations were performed on the optimized structures to obtain the relative energy changes along the proposed pathway.

In this work, metal atoms were placed at termination-top sites of pristine MXene surfaces to ensure a consistent comparison across different systems. Although hollow, bridge, and defect/vacancy sites may also act as favorable anchoring centers in MXenes, these configurations were not explicitly considered. Therefore, the present results should be interpreted within an idealized, low-defect surface model.

### 2.2. Energy Analysis

The stability and aggregation resistance of the MXene-supported single-atom catalyst systems were evaluated using the binding energy (*E*_b_), cohesive energy (*E*_c_), and bulk-referenced formation energy ($E_{\mathrm{bulk}}^{f}$). The *E*_b_ was used to quantify the interaction strength between the isolated metal atom and the MXene surface, whereas the *E*_c_ was introduced to describe the intrinsic tendency of the corresponding metal atoms to aggregate into the bulk metallic phase. To further provide a thermodynamic criterion for evaluating the stability of isolated single atoms against metal aggregation, the $E_{\mathrm{bulk}}^{f}$ was calculated by *E*_b_ and *E*_c_. These energetic descriptors are defined as follows: Their evaluations are defined as follows:

(1) $$E_{b}=E_{catalyst}-E_{MXene}-E_{atom}$$

(2) $$E_{c}=E_{bulk}/N-E_{atom}$$

(3) $$E_{f}^{bulk}=E_{b}-E_{c}$$

where *E*_catalyst_, *E*_MXene_, and *E*_atom_ denote the energy of MXene single-atom catalysts, MXene, and a single atom, respectively. *E*_bulk_ is the total energy of the bulk crystal unit cell of the corresponding metal, and N is the number of metal atoms in the bulk unit cell. Under the present definition, *E*_c_ is negative and represents the energy change associated with the aggregation of isolated metal atoms into the corresponding bulk metallic phase.

The adsorption energy (*E*_a_) was employed to evaluate the interaction between probe molecules and surface acidic sites. The adsorption energy was calculated as

(4) $$E_{a}=E_{total}-E_{catalyst}-E_{NH_{3}}$$

where *E*_total_, *E*_catalyst_, and $E_{NH_{3}}$ denote the total energy of the adsorption system, the MXene single-atom catalysts, and NH_3_, respectively.

## 3. Results and Discussion

### 3.1. Energetic Stability and Adsorption Properties of SACs

The total energies used for calculating *E*_b_, *E*_c_, $E_{\mathrm{bulk}}^{f}$, and *E*_a_ are listed in Tables S1 and S2, and the corresponding calculated values are summarized in Table 1 to evaluate the anchoring strength, aggregation resistance, and Lewis acidity of MXene-supported single-atom sites. The *E*_b_ values reflect the direct metal–support interaction between the isolated metal atom and the MXene surface. A more negative *E*_b_ generally indicates stronger interfacial interaction and more favorable thermodynamic anchoring of the single atom. However, because *E*_b_ is referenced to an isolated metal atom, it cannot by itself provide a realistic assessment of the aggregation tendency of metal atoms. Therefore, $E_{\mathrm{bulk}}^{f}$, which incorporates the cohesive energy of the corresponding bulk metal, was further used to evaluate the thermodynamic stability of isolated single atoms relative to metal aggregation. According to the present definition, a negative $E_{\mathrm{bulk}}^{f}$ indicates that anchoring of the metal atom on the MXene surface is thermodynamically more favorable than the formation of the corresponding bulk metal phase, suggesting an anti-aggregation tendency. In contrast, a positive $E_{\mathrm{bulk}}^{f}$ indicates that metal aggregation is thermodynamically preferred. Therefore, the combined analysis of *E*_b_, *E*_c_, and $E_{\mathrm{bulk}}^{f}$ provides a more reliable criterion for screening thermodynamically feasible MXene-supported single-atom sites.

Considering that -O and -OH-terminated surfaces are generally considered more representative of experimentally treated Ti_3_C_2_T_x_ MXenes after post-treatment, the detailed electronic structure analysis and acidity discussion in this work were focused on these two surface terminations.

As shown in Table 1, the stability of the investigated single-atom sites is strongly dependent on both the metal species and the surface termination of Ti_3_C_2_T_x_. For the O-terminated Ti_3_C_2_O_2_ surface, Ti exhibits the strongest interaction with the MXene substrate, with an *E*_b_ value of −6.07 eV. Its negative $E_{\mathrm{bulk}}^{f}$ value of −0.75 eV further indicates that Ti single atoms are thermodynamically more favorable on Ti_3_C_2_O_2_ than in the Ti bulk phase. Sn/Ti_3_C_2_O_2_ also shows a negative $E_{\mathrm{bulk}}^{f}$ value of −0.21 eV, suggesting that Sn single atoms can be stabilized on the Ti_3_C_2_O_2_ surface, although the thermodynamic driving force is weaker than that of Ti/Ti_3_C_2_O_2_. Ga/Ti_3_C_2_O_2_ gives a slightly negative $E_{\mathrm{bulk}}^{f}$ value of −0.17 eV, indicating only marginal thermodynamic stability. In contrast, Ni, Fe, and Ru on Ti_3_C_2_O_2_ exhibit positive $E_{\mathrm{bulk}}^{f}$ values of 2.40, 4.89, and 3.82 eV, respectively, implying that these metal atoms are more prone to aggregation into their corresponding bulk phases at the considered top-site configuration.

Compared with the O-terminated Ti_3_C_2_O_2_ surface, the hydroxylated Ti_3_C_2_(OH)_2_ surface exhibits significantly weaker anchoring capability for the investigated metal atoms. The *E*_b_ values of Ti, Ga, Sn, and Ru on Ti_3_C_2_(OH)_2_ are −2.70, −1.45, −2.71, and −1.89 eV, respectively, which are considerably less negative than those on the O-terminated surface. Correspondingly, all these systems exhibit positive $E_{\mathrm{bulk}}^{f}$ values, indicating that the top site of Ti_3_C_2_(OH)_2_ is not thermodynamically favorable for stabilizing isolated metal atoms against aggregation. In addition, Fe and Ni single atoms could not be stabilized at the selected top-site configuration on Ti_3_C_2_(OH)_2_, as no physically meaningful optimized structures were obtained. These results suggest that the OH- termination weakens metal-support interactions, probably due to the chemical saturation of surface coordination sites.

The Lewis acidity of the thermodynamically feasible single-atom sites was further evaluated by NH_3_ adsorption. Ti/Ti_3_C_2_O_2_ exhibits a relatively strong NH_3_ adsorption energy of −2.55 eV, indicating pronounced Lewis acidity at the Ti single-atom site. In contrast, Sn/Ti_3_C_2_O_2_ shows weaker NH_3_ adsorption with an *E*_a_ value of −1.44 eV, corresponding to a comparatively weaker Lewis acidic site. For systems with unfavorable thermodynamic stability or unstable adsorption configurations, the NH_3_ adsorption energies were not included in the quantitative acidity comparison.

Overall, Ti/Ti_3_C_2_O_2_ and Sn/Ti_3_C_2_O_2_ were selected as representative models for subsequent electronic structure and catalytic mechanism analyses. Ti/Ti_3_C_2_O_2_ represents a strongly anchored and more acidic single-atom site, whereas Sn/Ti_3_C_2_O_2_ provides a moderately stable single-atom site with weaker Lewis acidity.

To further understand the structural response upon NH_3_ adsorption, key bond lengths before and after adsorption were analyzed (Table 2). For both Ti/Ti_3_C_2_O_2_ and Sn/Ti_3_C_2_O_2_ systems, the M-O bond lengths remain within a narrow range after NH_3_ adsorption, indicating that the anchored single-atom sites maintain structural stability during the adsorption process. Meanwhile, the formation of M-N bonds (2.247 Å for Ti-N and 2.648 Å for Sn-N) confirms the direct coordination interaction between NH_3_ and the single-atom centers. Compared with Sn/Ti_3_C_2_O_2_, the shorter Ti-N distance suggests a stronger interaction between NH_3_ and the Ti active site, which is consistent with the stronger NH_3_ adsorption behavior observed in the adsorption energy analysis. In addition, only minor variations are observed for the Ti-O and Ti-C bonds in the MXene framework, indicating that NH_3_ adsorption mainly induces local structural adjustment around the active site rather than significant distortion of the MXene substrate.

### 3.2. Electronic Structure Analysis

#### 3.2.1. Density of States Analysis and Partial Density of States Analysis

**Figure 2:**
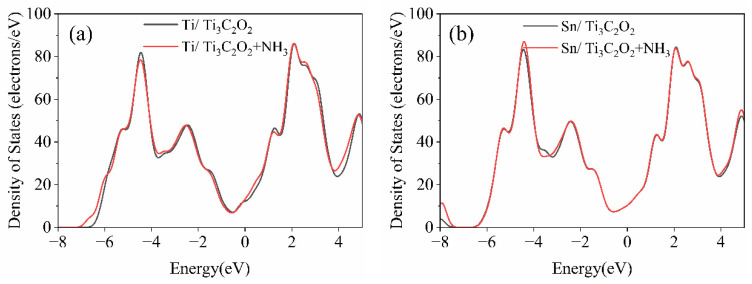
Density of states of representative MXene-supported single-atom systems before (**a**) and after NH_3_ adsorption (**b**).

To rationalize the termination- and metal-dependent probe adsorption behaviors, the DOS was analyzed for representative systems before and after NH_3_ adsorption (Figure 2). DOS calculation provides information on the number of electron or hole states per unit volume at a given energy level, which helps to verify the direct band gap structure and electronic properties [16,17]. Although Ti_3_C_2_-based MXenes generally exhibit metallic characteristics, the introduction of isolated single atoms and subsequent NH_3_ coordination induce distinct termination-dependent redistributions of electronic states near the Fermi level. Such changes directly reflect the charge-transfer capability and polarization response of the active sites, which are closely related to Lewis acidity. The DOS results show that NH_3_ adsorption does not significantly alter the overall electronic structure of Ti/Ti_3_C_2_O_2_ and Sn/Ti_3_C_2_O_2_, as the main DOS profiles before and after adsorption remain generally similar. This indicates that NH_3_ adsorption mainly induces local electronic perturbation around the metal anchoring sites rather than causing obvious reconstruction of the whole MXene electronic framework.

**Figure 3:**
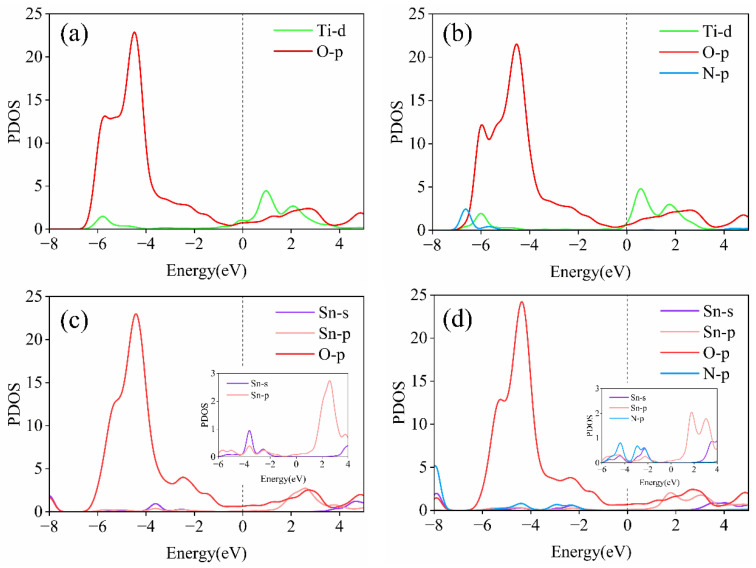
(**a**) PDOS of Ti/Ti_3_C_2_O_2_. (**b**) PDOS of Ti/Ti_3_C_2_O_2_ + NH_3_. (**c**) PDOS of Sn/Ti_3_C_2_O_2_. (**d**) PDOS of Sn/Ti_3_C_2_O_2_ + NH_3_.

To further clarify the local electronic interaction, PDOS analysis was performed (Figure 3). For Ti/Ti_3_C_2_O_2_, the Ti-d states are distributed near the Fermi level and show noticeable overlap with the O-p states of the surface termination, indicating electronic coupling between the anchored Ti atom and the O-terminated MXene surface. After NH_3_ adsorption, the PDOS profile around the Fermi level changes to some extent, suggesting that NH_3_ interacts with the Ti-centered site through local orbital interaction. This is consistent with the relatively strong NH_3_ adsorption behavior of Ti/Ti_3_C_2_O_2_.

For Sn/Ti_3_C_2_O_2_, the PDOS before and after NH_3_ adsorption shows a smaller change near the Fermi level compared with Ti/Ti_3_C_2_O_2_. Although Sn-related states contribute to the electronic structure, the overlap between the active metal states and surface O-p states is less pronounced. This suggests a weaker local electronic interaction between NH_3_ and the Sn-centered site. Therefore, the PDOS results support that the Lewis acidity of the single-atom sites is closely related to the local metal-termination electronic coupling and the electronic states available near the Fermi level.

#### 3.2.2. Mulliken Charge Transfer and Differential Charge Density

To further corroborate the acidity trends inferred from NH_3_ adsorption energies and electronic structure analysis, charge transfer upon NH_3_ adsorption was examined using a combination of Mulliken charge analysis and charge-density difference (Δρ) calculations. These two approaches provide complementary insights: Mulliken charges describe the overall direction and relative magnitude of electron transfer, while Δρ maps visualize the spatial redistribution of electrons at the probe-site interface [18,19].

**Figure 4:**
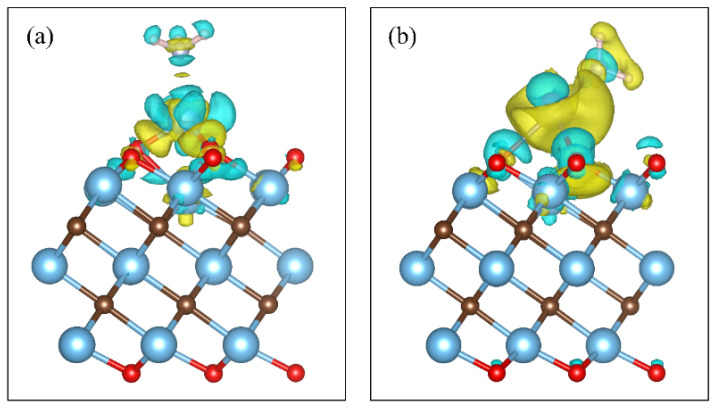
Charge density difference of NH_3_ adsorption on representative MXene-supported single-atom sites: (**a**) Ti/Ti_3_C_2_O_2_ + NH_3_. (**b**) Sn/Ti_3_C_2_O_2_ + NH_3_. Note: yellow indicates an increase in charge density; blue indicates a decrease in charge density.

Consistent with the DOS analysis, NH_3_ adsorption induces evident charge redistribution around the single-atom active centers and adjacent surface atoms. For the O-terminated systems, electron accumulation and depletion regions are mainly localized at the metal–NH_3_ interface, indicating direct electronic interaction between the adsorbate and the anchored metal atoms (Figure 4a,b). For Ti/Ti_3_C_2_O_2_ (Figure 4a), the charge redistribution is primarily concentrated around the Ti center and neighboring O atoms, suggesting localized electronic polarization within the Ti-O-MXene framework upon NH_3_ adsorption. In contrast, Sn/Ti_3_C_2_O_2_ (Figure 4b) exhibits a more spatially extended charge redistribution region, indicating a broader electronic perturbation involving both the anchored Sn atom and the surrounding surface environment. These observations are consistent with the PDOS results, which reveal changes in the local electronic states after NH_3_ adsorption. Overall, the charge density difference analysis confirms that NH_3_ adsorption is accompanied by noticeable electronic interaction between the probe molecule and the single-atom sites, providing further evidence for the termination-regulated electronic properties of MXene-supported metal centers.

### 3.3. Validation of the Identified Lewis Acidic Site

To preliminarily assess whether the Lewis acidic site identified through the thermodynamic and electronic structure analyses may facilitate biomass-derived molecular activation, cellulose was selected as a representative biomass model, and the relative energy changes along a proposed reaction pathway were examined. The reported values were obtained from selected optimized configurations and are therefore discussed as overall relative energy requirements rather than kinetic activation barriers.

**Figure 5:**
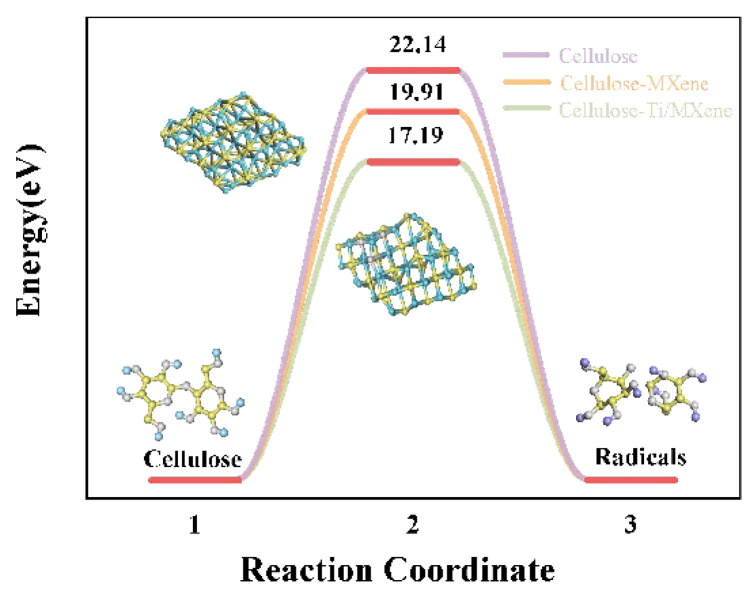
Proposed reaction pathways and relative energy profiles for cellulose-derived conversion in the uncatalyzed, Ti_3_C_2_O_2_, and Ti/Ti_3_C_2_O_2_ systems.

As shown in Figure 5, the uncatalyzed pathway exhibits the highest relative energy requirement of 22.14 eV, indicating that the proposed conversion of cellulose-derived species into radical intermediates is energetically demanding. In the presence of Ti_3_C_2_O_2_, the corresponding value decreases to 19.91 eV, suggesting that the O-terminated MXene surface modifies the energetics of the proposed transformation. The Ti/Ti_3_C_2_O_2_ system further decreases the relative energy requirement to 17.19 eV, corresponding to reductions of 22.4% and 13.7% relative to the uncatalyzed and pristine Ti_3_C_2_O_2_ systems, respectively. The significantly lower relative energy demonstrates that the introduction of isolated Ti sites effectively enhances the catalytic activity of the MXene surface.

The observed energetic trend is qualitatively consistent with the preceding stability, acidity, and electronic structure analyses. The negative bulk-referenced formation energy supports the thermodynamic feasibility of stabilizing isolated Ti atoms on Ti_3_C_2_O_2_, while the strong NH_3_ adsorption indicates pronounced Lewis acidity at the Ti center. The DOS and PDOS results reveal substantial electronic interactions between the anchored Ti atom and the O-terminated MXene substrate, and the charge-density difference analysis further shows pronounced electronic redistribution around the Ti site. These characteristics may contribute to the lower relative energy requirement obtained for Ti/Ti_3_C_2_O_2_.

Overall, the relative energy analysis provides qualitative support for the proposed relationship between the electronic characteristics of the Ti single-atom site and its potential role in biomass-derived molecular activation. The results should be interpreted as a preliminary energetic comparison among the investigated systems rather than as a rigorous kinetic evaluation.

## 4. Conclusions

In this work, the Lewis acidity of MXene-supported single-atom sites was systematically investigated by coupling surface functional groups with metal identity. Using Ti_3_C_2_-based MXenes as model substrates, formation energy and NH_3_ adsorption energy were employed to screen thermodynamically feasible and acidic single-atom configurations. Among the investigated systems, Ti/Ti_3_C_2_O_2_ was identified as a representative acidic single-atom site. Electronic structure analyses demonstrate that enhanced acidity originates from termination-induced electronic polarization and effective charge redistribution at the metal-termination interface. The combined analysis of adsorption energetics and electronic structure further indicates that sites with stronger electron-accepting capability exhibit an enhanced ability to induce bond polarization in oxygen-containing species, which is critical for key elementary steps in biomass conversion processes. Based on these findings, Ti/Ti_3_C_2_O_2_ was identified as a promising candidate for catalytic pyrolysis reactions toward biomass-based chemical formation.

## Figures and Tables

**Table 1:** Calculated formation energies and adsorption energies of the systems considered in this study.

Metal		Ti_3_C_2_O_2_			Ti_3_C_2_(OH)_2_		
	***E*** **_c_**	***E*** **_b_**	***E*** **_a_**	** $E_{\mathrm{bulk}}^{f}$ **	***E*** **_b_**	***E*** **_a_**	** $E_{\mathrm{bulk}}^{f}$ **
Ti	−5.33	−6.07	−2.55	−0.75	−2.70	/	2.63
Ni	−4.65	−2.26	/	2.40	/	/	/
Fe	−4.97	−0.08	/	4.89	/	/	/
Ga	−2.81	−2.98	/	−0.17	−1.45	/	1.36
Sn	−3.68	−3.89	−1.44	−0.21	−2.71	/	0.97
Ru	−6.64	−2.82	/	3.82	−1.89	/	4.76

Note: / indicates that no physically meaningful optimized structure or reliable NH_3_ adsorption configuration was obtained; therefore, the corresponding value was not included in the quantitative analysis.

**Table 2:** Key bond lengths (Å) for Ti/Ti_3_C_2_O_2_ and Sn/Ti_3_C_2_O_2_ before and after NH_3_ adsorption.

	d_M-O_/Å	d_Ti-O_/Å	d_Ti-C_/Å	d_N-H_/Å	d_M-N_/Å
NH_3_	/	/	/	1.025	/
Ti/Ti_3_C_2_O_2_	1.862	2.252	2.172	/	/
Ti/Ti_3_C_2_O_2_ + NH_3_	1.827	2.327	2.166	1.030	2.247
Sn/Ti_3_C_2_O_2_	2.239	2.143	2.179	/	/
Sn/Ti_3_C_2_O_2_ + NH_3_	2.186	2.067	2.184	1.026	2.648

Note: d_M–O_: distance between the single atom (Ti or Sn) and the nearest surface O atom. d_Ti–O_: distance between surface O and the nearest Ti atom in the MXene lattice. d_Ti–C_: distance between surface Ti and neighboring C atom. d_N–H_: N–H bond length in NH_3_. d_M–N_: distance between the single atom and the N atom of adsorbed NH_3_.

## Data Availability

The original contributions presented in this study are included in the article. Further inquiries can be directed to the corresponding authors.

## Supplementary Materials

The following supporting information can be downloaded at: https://www.mdpi.com/article/10.3390/nano16130800/s1, Table S1: Calculated $E_{\mathrm{TM}}$ and $E_{\mathrm{bulk}}/N$ values used for evaluating metal cohesive energies; Table S2: Total energies of MXene-supported single-atom catalysts and corresponding NH_3_-adsorbed systems.
