# Supplementary material for: Tuning Lewis Acidity in MXene-Supported Single-Atom Catalysts
Source: Nanomaterials (Basel). 2026 Jun 27;16(13):800. doi: 10.3390/nano16130800 (PMC13363295; doi:10.3390/nano16130800)
Supplement: Supplementary file 1 [file nanomaterials-16-00800-s001.zip › nanomaterials-4316196-supplementary.pdf]

nanomaterials

MDPI

Supplementary Materials

Tuning Lewis Acidity in MXene-Supported Single-Atom Catalysts

Weiqiang Sun [1], Tingting Zhou [2], Boyu Han [2], Hu Xu [3], Junqi Wang [2,*] and Bo Yu [4,*]

[1] School of Nuclear Science and Technology, Xi'an Jiaotong University, Xi'an 710049, China; sunweiqiang@xjtu.edu.cn
[2] School of Human Settlements and Civil Engineering, Xi'an Jiaotong University, Xi'an 710049, China; 372498563@stu.xjtu.edu.cn (T.Z.); hanboyu0036@stu.xjtu.edu.cn (B.H.)
[3] School of Physics, Xi'an Jiaotong University, Xi'an 710049, China; xu_hu@xjtu.edu.cn
[4] State Key Laboratory of Advanced Casting Technologies, Shenyang 110022, China
* Correspondence: wjq@xjtu.edu.cn (J.W.); yub@chinasrif.com (B.Y.)

Table S1. Calculated E_{TM} and E_{bulk}/N values used for evaluating metal cohesive energies.

Metal	E_{TM} (eV)	E_{bulk}/N (eV)
Ti	-1588.514	-1593.841
Ni	-1371.322	-1375.977
Fe	-857.970	-862.942
Ga	-2145.723	-2148.530
Sn	-2056.723	-2060.406
Ru	-2617.249	-2623.893

Note: TM denotes the single metal atom.

Table S2. Total energies of MXene-supported single-atom catalysts and corresponding NH_3-adsorbed systems.

Metal	$E_{TM\text{-}O}$ (eV)	$E_{TM-O+NH_3}$ (eV)	$E_{TM\text{-}OH}$ (eV)	$E_{TM-OH+NH_3}$ (eV)
	-53853.099		-54136.571	
Ti	-55447.687	-55774.451	-55727.781	-56055.798
Ni	-55226.681	-55553.650	/	/
Fe	-54711.151	-55039.874	/	/
Ga	-56001.802	-56327.012	-56283.740	/
Sn	-55913.716	-56239.373	-56196.003	-56521.029
Ru	-56473.171	-56800.188	-56755.707	/

Note: TM-O denotes the total energy of the single metal atom anchored on $Ti_3C_2O_2$ surface; $E_{TM\text{-}OH}$ denotes the total energy of the single metal atom anchored on $Ti_3C_2(OH)_2$ surface. E_{NH_3}: -324.213 (eV)

https://doi.org/10.3390/nano16130800
